# Big Data Approaches for the Analysis of Large-Scale fMRI Data Using Apache Spark and GPU Processing: A Demonstration on Resting-State fMRI Data from the Human Connectome Project

**DOI:** 10.3389/fnins.2015.00492

**Published:** 2016-01-06

**Authors:** Roland N. Boubela, Klaudius Kalcher, Wolfgang Huf, Christian Našel, Ewald Moser

**Affiliations:** ^1^Center for Medical Physics and Biomedical Engineering, Medical University of ViennaVienna, Austria; ^2^MR Centre of Excellence, Medical University of ViennaVienna, Austria; ^3^Department of Radiology, Tulln Hospital, Karl Landsteiner University of Health SciencesTulln, Austria; ^4^Brain Behaviour Laboratory, Department of Psychiatry, University of Pennsylvania Medical CenterPhiladelphia, PA, USA

**Keywords:** fMRI, big data analytics, distributed computing, graph analysis, Apache Spark, scalable architecture, machine learning, statistical computing

## Abstract

Technologies for scalable analysis of very large datasets have emerged in the domain of internet computing, but are still rarely used in neuroimaging despite the existence of data and research questions in need of efficient computation tools especially in fMRI. In this work, we present software tools for the application of Apache Spark and Graphics Processing Units (GPUs) to neuroimaging datasets, in particular providing distributed file input for 4D NIfTI fMRI datasets in Scala for use in an Apache Spark environment. Examples for using this Big Data platform in graph analysis of fMRI datasets are shown to illustrate how processing pipelines employing it can be developed. With more tools for the convenient integration of neuroimaging file formats and typical processing steps, big data technologies could find wider endorsement in the community, leading to a range of potentially useful applications especially in view of the current collaborative creation of a wealth of large data repositories including thousands of individual fMRI datasets.

## 1. Introduction

The pressure to continuously analyze fast growing datasets has led internet companies to engage in the development of specialized tools for this new field of Big Data analysis, at first strongly focused on the specific data structures used by their applications, but increasingly taking more generalized forms. One of the most fundamental developments in this area is Google's MapReduce paradigm (Dean and Ghemawat, 2004), designed for efficient distributed computations on datasets too large to fit on a single machine, which are instead stored in a distributed file system in a cluster environment. The computation concept behind MapReduce is to use the individual cluster nodes where the data are stored as efficiently as possible by transfering as much of the computation as possible to the individual storage nodes instead of transfering their data to a designated compute node, and only perform subsequent aggregation steps of the computation to master compute nodes. Thus, there exists a strong link between the distributed data storage and the computation. For example, Apache's open source implementation of the paradigm consists of Hadoop, the implementation of the actual MapReduce computation engine, and the Hadoop Distributed File System (HDFS) for data storage. The Hadoop ecosystem is further complemented by a variety of toolkits for specialized applications like machine learning.

The principles of the MapReduce paradigm can best be illustrated using the distributed algorithm for counting the number of occurrences of words in large documents, the canonical example for MapReduce computations. As the name suggests, these computations consist of two steps, termed Map and Reduce, with Map being performed on each node separately, and the Reduce step computed on a central node, aggregating the individual Map results. In the word count example, the Map step would consist in generating, for each part of the document stored on the distributed file system, a set of keys and values, with words being the keys and the number of occurrences of each word being the associated value. The Reduce step would then aggregate these partial results by building the sum of all values from all individual nodes associated with each word, thus gaining the overall number of occurrences of this word in the entirety of the dataset.

While the approach proves to be flexible enough for a wide range of computations, this brief description should also make it apparent that not all kinds of computations can be performed in this way. For example, many data analysis applications, like iterative machine learning algorithms, need to access data multiple times, which would be very inefficient if implemented in pure MapReduce terms. Addressing this issue and providing a more general framework for distributed computations on large datasets was the main motivation behind the introduction of the Spark framework (Zaharia et al., 2012; The Apache Software Foundation, 2015). The counterpart of data stored in the Hadoop distributed filesystem in the Spark framework are so-called resilient distributed datasets (RDD), which, unlike files in the HDFS, can be held entirely in memory if space allows (and cached to disk if memory is not sufficient), and provide a high-level interface for the programmer. The details of the distributed storage and computation on this distributed dataset are thus abstracted, making the writing of distributed code much easier in practice. Furthermore, Spark encompasses higher-level libraries for many applications including machine learning (MLlib) and graph analysis (GraphX), further facilitating the development of analyses in these specific domains. Spark can be used interactively from a Scala shell or via its Application Programming Interface (API), with APIs existing for Scala, Java, python and, most recently, R. With Spark being written in Scala and the interactive shell being a Scala shell, the connection between Spark and Scala is the strongest, and the other languages' APIs do not yet have the full functionality of the Scala API; for example, there is no interface to many functions of GraphX in python, and the R API is currently only in an early stage of development.

A further approach to accelerating computations on large datasets by parallelization, though not directly related to the Big Data technologies in the stricter sense mentioned above, concerns optimization of computations on a single machine, where in particular the use of Graphics Processing Units (GPUs) can make an enormous difference in terms of computational efficiency and thus rendering possible the analysis of even larger datasets in a reasonable amount of time.

Both the big data frameworks and GPU acceleration can prove useful in the field of neuroimaging in general and functional MRI in particular, where increasing spatial and temporal resolutions as well as larger sample sizes lead to a rapid increase in the amount of data that needs to be processed in a typical study. GPU computing has been embraced not only to provide faster programs for standard algorithms (Eklund et al., 2014), but also to make some more complex analyses possible at all (Boubela et al., 2012; Eklund et al., 2012, 2013). Apart from such special tools, GPU acceleration has in some cases already be harnessed in standard neuroimaging data analysis libraries like, for example, in FSL (Jenkinson et al., 2012). In contrast to Big Data technologies in the narrower sense, however, these technologies do not scale arbitrarily, but are instead limited to the amount of data that can be held in memory on a single machine. But while GPUs have slowly been picked up by the neuroimaging community, the spread of Hadoop and Spark is more limited. In the context of the Human Connectome Project, Marcus et al. (2011) describe the infrastructure for the storage and exploration of such a large dataset, but do not employ big data tools for efficient analyses on the whole dataset of 1400 subjects. Only two published papers have yet used them in the field of neuroimaging: Wang et al. (2013) used Hadoop to use random forests for machine learning on a large imaging genetics dataset, and Freeman et al. (2014) provide an analysis framework based on Apache Spark and highlight applications for two-photon imaging and light-sheet imaging data.

The dearth in this domain is all the more surprising in view of the emergence of a number of data sharing initiatives and large-scale data acquisition projects covering a wide array of topics in human neuroimaging (Biswal et al., 2010; ADHD-200 Consortium, 2012; Nooner et al., 2012; Assaf et al., 2013; Jiang, 2013; Mennes et al., 2013; Van Essen et al., 2013; Satterthwaite et al., 2016). Certainly, the opportunities offered by the availability of neuroimaging data from a large number of subjects are coming with some challenges (Akil et al., 2011). As has previously been noted, the sheer size of the datasets and their complexity require new approaches to harvest the full benefit of “human neuroimaging as a big data science” (Van Horn and Toga, 2014). For example, Zuo et al. (2012), when computing network centrality measures at a voxel-wise level, resampled all datasets to a 4 mm (isotropic) resolution and stated two reasons for this choice. The first reason is the average resolution of the datasets available from the 1000 Functional Connectomes dataset in the largest voxel dimension, which was not much below 4 mm, leading to the conclusion that using a higher resolution might not be worth the effort on this dataset. The second stated reason was the computational demands that a higher resolution would require: while the voxelwise network at a 4 mm resolution had 22,387 nodes, this number would increase to 42,835 when using a 3 mm resolution. Since then, even higher resolutions than 3 mm have become more and more common— the Human Connectome Project dataset for example uses isotropic 2 mm voxels—and the need to address the computational demands that accompany this increase in data size becomes obvious.

Still, while large-scale data repositories could provide a good model on how to use big data technologies in human neuroimaging, they have not yet been explored with these methods. One reason for the neuroimaging community not embracing big data tools more readily might be the lack of reasonably efficient I/O from (and, to a lesser extent, to) standard neuroimaging file formats like NIfTI. Removing this barrier of entry might open the way to a variety of analysis tools that could then be directly applied to datasets of practically arbitrary size. While the range of tools that can currently be applied to large datasets is limited to computationally relatively simple methods like regression, scaling the computation power using clusters can extend this to more complex machine learning and graph mining algorithms, including methods without closed form solution that need to be solved iteratively. Another research area where computationally intensive methods might prove useful is the investigation of reliability and reproducibility of neuroimaging methods as reviewed by Zuo and Xing (2014), who also note that easing the computational demand by aggregation, e.g., averaging the signal from multiple voxels based on anatomical structure, leads to difficulties in the reliability and interpretation of derived results, and strongly encourage voxel-wise analysis for the evaluation of the functional architecture of the brain. The Consortium for Reliability and Reproducibility in particular has gathered a large dataset of over 5000 resting-state fMRI measurements to this end (Zuo et al., 2014), and proposes a number of computational tools for use on this database, yet these do not currently include big data tools.

## 2. Testing platform and data

### 2.1. Computing environment

The computation of the connectivity matrices based on Pearson correlation were performed on a server running Ubuntu Linux (version 12.04) equipped with 192 GB random access memory (RAM), two Intel Xeon X5690 processors and four Nvidia Tesla C2070 GPUs. As an aside, it should be noted that while these GPUs are somewhat dated, they already have full support for double precision computations; while modern GPUs no longer have issues with double precision computation, older generations (with compute capability < 2.0, corresponding approximately to models developed before 2011) might be slow or unable to perform anything but single precision computations. The linear algebra operations on the GPUs were accessed using CUDA 6.0 and integrated in R (Boubela et al., 2012; R Core Team, 2014). Spark was used via the Scala shell and API for the practical reasons discussed above. OpenBLAS version 0.2.14 was compiled and installed for the Apache Spark compute nodes to enable these machine optimzed libraries to be used by Spark's linear algebra functionality in MLlib. Further, R uses the OpenBLAS implementation of the singular value decomposition (SVD) for performance comparison purposes.

The cluster running Apache Spark 1.5.1 consists of ten Sun Fire X2270 servers using Ubuntu Linux (version 14.04) with 48 GB RAM and two Intel Xeon X5550 processors. Additionally, each server uses three 500 GB hard disk drives (HDD) as local disk space for the Apache Spark framework. Beside a standard 1 GBit ethernet connection, the cluster nodes are connected via the IP over Infiniband protocol on QDR Infiniband hardware.

### 2.2. Subjects

To test the methods described, 200 sample datasets from the Human Connectome Project (Van Essen et al., 2013) were downloaded from the project repository and used for example analyses. In this study, only the resting-state fMRI data were used, though the methods are not limited to this type of data.

### 2.3. Source code

All code presented in this work can be found in the github repository https://github.com/rboubela/biananes.

## 3. Human connectome project data analysis

### 3.1. NIfTI file input for fMRI

**Figure 1:**
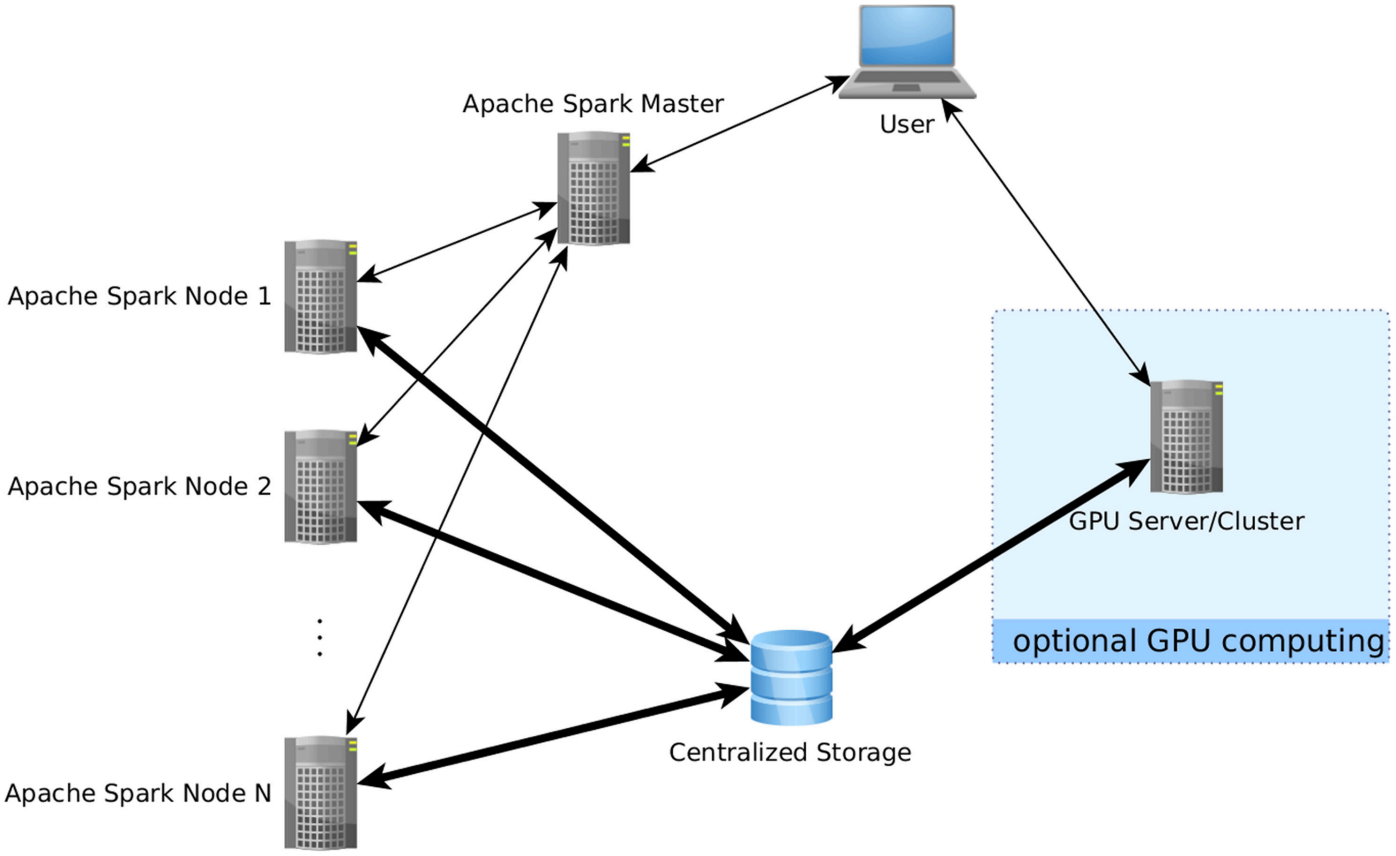
**Data flow using the proposed analysis methods**. Bold arrows represent intensive data flow, the other arrows represent communication of control commands. In this example, parts of the computations have been performed on a separate GPU computing server which was not part of the Apache Spark cluster. The use of a centralized data storage facilitates the integration of all steps into a comprehensive pipeline, as the fMRI data is loaded directly from there onto the GPU server, who then writes the results as edgelist back on the storage to be directly readable by Apache Spark. Note that storing data directly on the compute nodes is also possible as an alternative if issues related to data transfer speed are encountered.

One of the most basic obstacles to using Apache Spark for fMRI datasets is the lack of an efficient file input function able to process any file formats usually used in this field like NIfTI-1. Of course, file readers in Java, python or R exist which could be used when using Spark from their respective API, and the Java file reader could be used in Scala (and thus also in the Scala shell), but none of those file readers is suited for the distributed environment. For this, a distributed file reader for fMRI data was implemented in Scala and C which reads 4D NIfTI files in parallel on multiple nodes, with each node reading a different set of the image's volumes, and gathers the results into an RDD in the Spark environment. To avoid unnecessary overhead, a brain mask can be used to restrict reading to in-brain voxels; the brain mask must also be available as a NIfTI file and will be applied to all volumes of the 4D NIfTI file to be read. Files can be read from local harddisks on the nodes or via the network file system (NFS) protocol from a centralized storage accessible to the compute nodes (see Figure 1). While in principle, the former method is faster than reading the files over the network, reading the input data is rarely the computational bottleneck in fMRI data analysis, and thus reading the input files even from the same common network storage device is efficient enough while typically being much more convenient. Nonetheless, for situations where fast file access over the network is not available, or if local storage is prefered for other reasons, the reader also allows for reading NIfTI input from local harddisks, in which case the NIfTI input file(s) must be available on all nodes under the same path.

**Figure 2:**
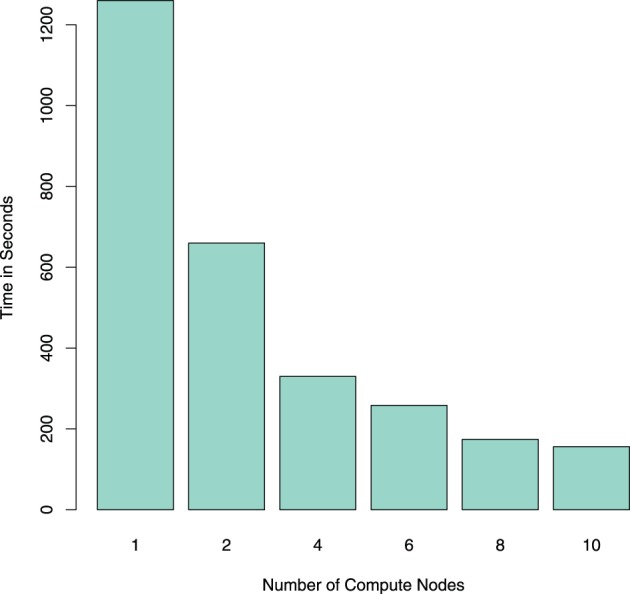
**Comparing the runtime (in seconds) for reading one resting-state fMRI dataset using *NiftiImageReadMasked* on an Apache Spark cluster with different numbers of compute nodes**. Note that the reduction in computation time scales almost with 1/n, n being the number of nodes in the cluster.

In Spark, the voxelwise timeseries data is stored in the columns of a *RowMatrix* object. This type of object is the most commonly used in the interface of the Apache Spark machine learning library *MLlib* for the distributed handling of large numerical matrices. For example, SVD or principal component analysis (PCA) can be applied directly on this *RowMatrix*, which in turn can be the basis for various further statistical analyses like independent component analysis (ICA). Column similarities based on the cosine similarity can also be computed efficiently on a *RowMatrix* in Spark (Zadeh and Goel, 2012; Zadeh and Carlsson, 2013). Examples for using the data input function are shown in code listings 1 and 2 for single-subject and group data import, respectively, and the runtime of the NIfTI reader is shown in Figure 2. To exemplify possible linear algebra computations, a call for the SVD computation from *MLlib* is shown at the end of code listing 2. It should be noted that while this toy example demonstrates that using *MLlib* functions is very straightforward and easy, it would not make much sense from a computational point of view in this particular case: on four nodes, the computation of 10 singular values and vectors takes 604 s, and the computation of 100 singular values and vectors takes 2700 s; the same values can be computed on a single one of those nodes using *svd* in R with OpenBLAS as linear algebra backend in 118 and 126 s, respectively. Using Spark for linear algebra computations seems only sensible if the size of input data precludes the use of standard optimized linear algebra packages like OpenBLAS. The examples that follow will thus focus on more data-intensive problems like graph mining, where even single-subject analysis can involve the handling of very large datasets.

Listing 1: Reading a single-subject fMRI dataset


  **import** org.biananes.io.NiftiTools
  
  **val** hcp_root = sys.env(“HCP_ROOT”)
  **val** func = “/MNINonLinear/Results/rfMRI_REST1_RL/rfMRI_REST1_RL.nii.gz”
  **val** mask = “/MNINonLinear/Results/rfMRI_REST1_RL/brainmask_fs.2.nii.gz”
  **val** template = “/usr/share/data/fsl-mni152-templates/MNI152lin_T1_2mm.nii.gz”
  
  **val** img_file = hcp_root + “167743” + func_file
  **val** mask_file = hcp_root + “167743” + mask
  
  **val** mat = NiftiImageReadMasked(img_file, mask_file, sc)
 


Listing 2: Reading a group of subjects storing the data in one big group matrix and compute SVD on this matrix


  **val** subjects = sc.textFile(hcp_root + “subjectIDs.txt”)
  
  **val** input_files = subjects.map{ subject **=>**
        **new** Tuple2(**new** String(hcp_root + subject + func)
             ,
          template) }.collect
  
  **val** group_matrix = input_files.map{
    f **=>** NiftiImageReadMasked(f._1, f._2, sc) }.reduce((m1, m2) **=>** **new** RowMatrix(m1.rows.union(m2.rows)))
  
  **val** svd_result = group_matrix.computeSVD(1000)
 


### 3.2. GPU connectivity matrix

**Figure 3:**
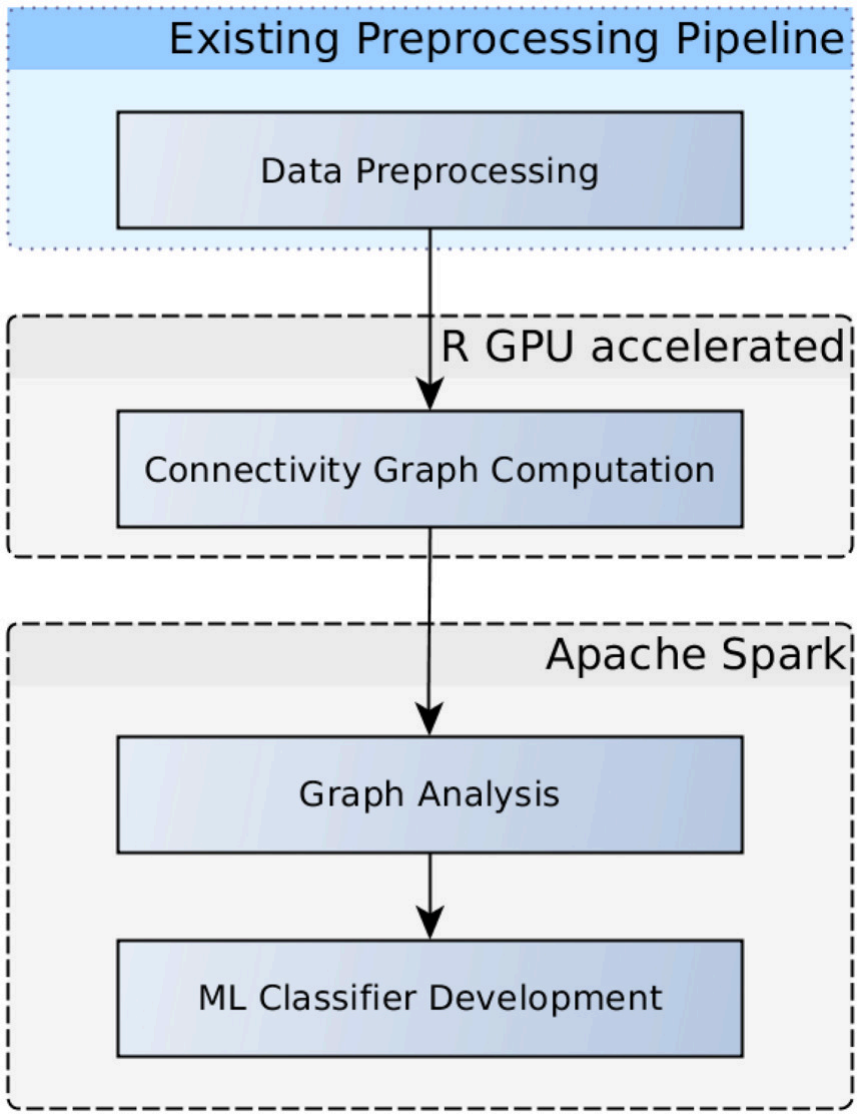
**Flowchart depicting an examplary analysis workflow**. Graphs based on voxelwise functional connectivity can be computed using the GPU accelerated R package. Graph measures using graph theory results can be extracted in Apache Spark, subsequently, these measures can be fed into the development of machine learning classifiers.

A more common similarity measure that can be used to compare voxel time series is the Pearson correlation coefficient, which is often used as functional connectivity measure in fMRI. Beside visualization of these connectivity patterns themselves, this measure can also be used in further analyses including machine learning (Eickhoff et al., 2016) or graph analyses (Craddock et al., 2015; Kalcher et al., 2015), as illustrated in the workflow diagram in Figure 3. In contrast to the above mentioned cosine similarity, Pearson correlation coefficients are simple linear algebra computations that can be computed by the arithmetic units on GPUs in a highly parallelized way, making it a viable application for GPU acceleration. Larger matrices might exceed the memory available on a GPU, however, but this problem can be addressed by tiling the input matrices in a way to separately compute submatrices of the result and subsequently concatenating the parts to form the complete matrix. In the case of the Human Connectome Project data, the voxelwise correlation matrix in the original resolution of all in-brain voxels (228200 ± 2589 voxels) for one subject takes up ~390 GB, which is divided into 91 tiles of 4.2 GB each (the rest of the GPU RAM is used up by the input needed to compute the respective tile).

**Figure 4:**
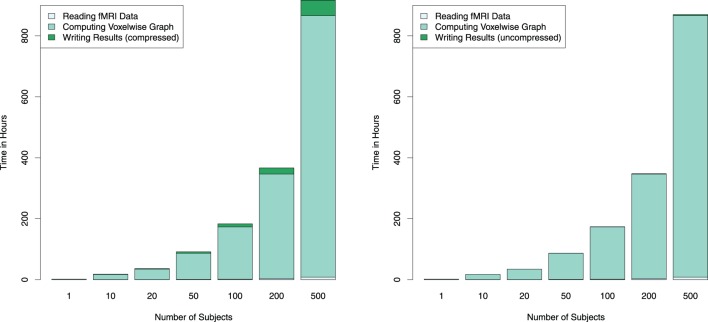
**Estimated combined computation times for reading the fMRI data, computing the connectivity graph and writing the thresholded (and thus sparse) connectivity matrix to compressed (left) and uncompressed (right) RData files are depicted for different numbers of subjects on a single GPU**. Since computation time depends linearly on the number of subjects, computation time for larger numbers of subjects are estimated using average per-subject computing times measured from 200 subjects. By employing multiple GPUs the runtime can be reduced linearly; for example, using four GPUs instead of one for computing 500 subject's connectivity graph would reduce the computation time from about 36 days down to 9.

The resulting correlation/connectivity matrix can be thresholded to obtain an adjacency matrix for a graph, with different options being available for the choice of a correlation threshold. For the estimation of the runtime for multiple subjects as shown in Figure 4, the matrix was thresholded at absolute values of 0.6 of the correlation coefficients. Subsequently, these sparse matrices were saved to RData files for further usage. (Note that since different fMRI datasets can be rather heterogeneous, it is in general more advisable to use an automated selection of a correlation threshold to achieve a certain edge density in the graph, for example defined by the value of *S* = log *E* ∕ log *K*, with *E* being the number of edges and *K* the average node degree.)

### 3.3. Graph analysis in apache spark

**Figure 5:**
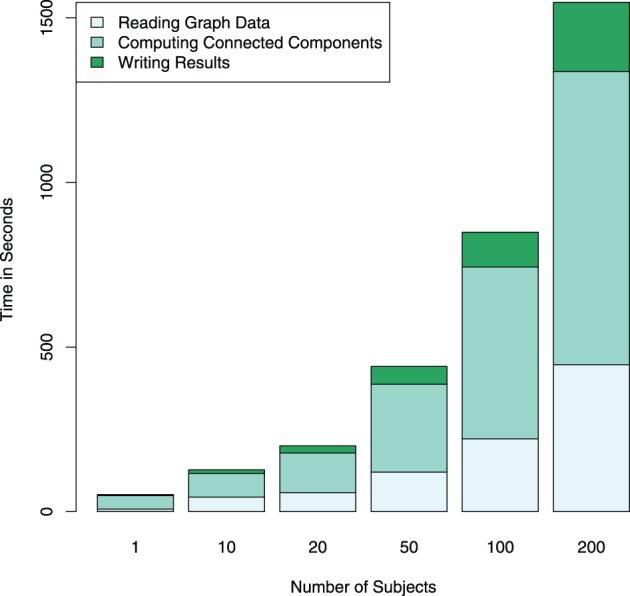
**Computation times for reading and writing the graph data in addition to computing connected components for a different number of subjects is shown performed on an Apache Spark cluster using four compute nodes**. The largest part of the computation time is spent on the graph computations themselves. Note that the computational complexity of the search for connected components is relatively low (*O(n)*), in the case of more complex computations, the proportion of the total computation time spent with data I/O further decreases.

**Figure 6:**
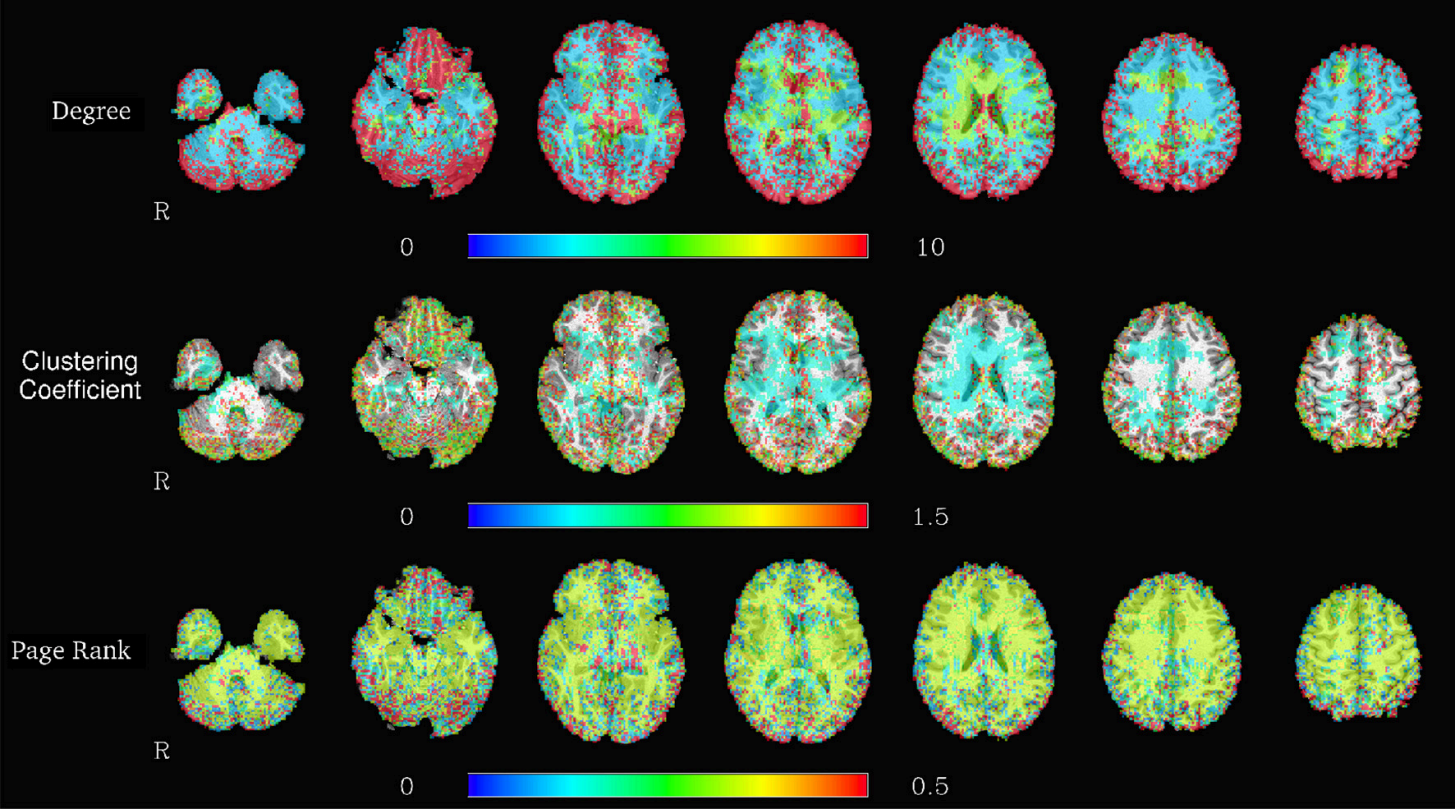
**Spatial distribution of node degrees (top), local clustering coefficients (center), and PageRank (bottom) at a voxelwise level for one representative subject, using the graph based on the correlation map thresholded at 0.6**.

The Apache Spark framework contains the *GraphX* library for the efficient development of distributed and scalable graph algorithms. A graph object from this library can be constructed from a variety of different inputs, including cosine similarities computed from the *RowMatrix* object or by directly reading a comma separated value (CSV) file containing a list of edges. Graphs defined using this library are represented in the Spark environment as two RDDs, one containing the vertices and the other the edges, in order to allow for distributed computations on the graph. Code listing 3 shows an example of importing multiple graphs from individual subject graph edge lists, and computing and saving connected components in each of the graphs. The corresponding computation times are illustrated in Figure 5, and exemplary results from graph analyses are shown in Figure 6.

Listing 3: Reading connectivity graphs from text files; computing connected components and storing results on disk


  **import** org.apache.spark.graphx._
  
  **val** edgeListFiles = sc.textFile(hcp_root + “hcp_edgelist_files.txt”).collect
  **val** graphs = edgeListFiles.map { edgefile **=>** **new** Tuple2(edgefile, GraphLoader.edgeListFile(sc, edgefile, **false**)) }
  
  // *compute* *the* *connected* *components* *for* *all* *graphs*
  **val** allConnectedComponents = graphs.map { g **=>** **new** Tuple2(g._1, g._2.connectedComponents().vertices) }
  
  // *saving* *the* *results*
  **val** resfiles = allConnectedComponents.map{ cc **=>** {
    **val** file = cc._1.substring(0, 106) + “connected_components”
      cc._2.coalesce(1, **true**).saveAsTextFile(file)
      file }
      }
 


One of the main advantages of using *GraphX* for graph analyses in fMRI is that computations can be distributed very easily to allow for pooled analysis of large groups of subjects. The example in code listing 4 demonstrates this using the example of the computation of voxelwise local clustering coefficients for all single-subject graphs read in the previous code listing. Note how the parallelized computation for all subjects is achieved with only a single line of code, without the need for explicit commands for the parallelization.

Listing 4: Computing the local clustering coefficient for each voxel for all graphs


  **val** allClusteringCoeff = graphs.map { g **=>** **new** Tuple2(g._1, g._2.degrees.join(g._2.triangleCount.vertices).map{ **case** (id, (d, tc)) **=>** (id, tc / (d ^*^ (d - 1) / 2.0))})
    }
 


## 4. Discussion

Big Data technologies are not yet often employed in the analysis of neuroimaging data, though the emergence of large collaborative repositories especially in the field of fMRI provides an ideal environment for their application. One of the main reasons for the currently limited interest in these technologies by researchers in neuroimaging seems to be a comparatively high effort for a first entry into this domain, in particular in view of the lack of interfaces to the data formats typically used in the field. Here, we present a distributed NIfTI file reader written in Scala for Apache Spark and show applications that become possible with this framework, including graph analyses using *GraphX*. In addition, the computation of correlation matrices from fMRI time series was implemented to run on GPUs and optimized for the 4D structure of time series fMRI data.

Most Spark code was written in Scala, which is the preferred language for development in this framework at the moment. However, interfaces to different languages are available at various stages of maturity, including python and R, which are both commonly used for fMRI data analysis. Though using Spark via the API from one of those languages does not currently provide access to the full range of analysis tools available in the Scala API, adding wrappers for these languages into our package would be a valuable addition.

Transferring fMRI computations into a Big Data analysis framework like Spark offers the advantage of the direct availability of a range of tools optimized for particular problems. Two of the most notable applications here are machine learning and graph data analysis, provided by the the Spark libraries *MLlib* and *GraphX*, respectively. Both machine learning and graph analysis are rapidly growing subfields in the fMRI community (Bullmore and Sporns, 2009; Craddock et al., 2015), but the applications of these methods is often limited by the computational means available for tackling the comparatively complex calculations involved.

Apart from efficiency in the sense of computation speed, a second type of efficiency is just as important in practical research software development: efficiency in terms of development time. While parallelization tools are available in multiple programming languages at different levels, one of the advantages of Spark in this respect is the relative ease with which it allows for distributing computations in cluster environments even in an interactive shell. As shown in code listing 4, the details of the distribution of computations is hidden from the developer, allowing for easier programming compared to other tools requiring explicit parallization. Furthermore, ease of access could be further improved by convergence with open data pipelines as developed in the context of data sharing initiatives (Zuo et al., 2014; Xu et al., 2015), as the inclusion of big data tools into published analysis pipelines could help spread such tools to a wider community of researchers who might otherwise not investigate these opportunities.

Another important aspect of using a scalable platform is the ability to avoid buying and operating on premise computing equipment, but instead move data analysis and computation tasks to cloud service providers. As Freeman et al. (2014) have shown in their work, using large amount of quickly available cloud computing resources can conveniently be leveraged using the Spark Framework. For example, in addition to running the Spark Framework, the Amazon web services (AWS) cloud (as used by Freeman et al., 2014) also provides compute nodes with GPUs (https://aws.amazon.com/ec2/instance-types/), and therefore, could also be employed for the GPU accelerated computation of connectivity graphs as proposed herein.

It is probably the difficulty of climbing the first steep learning curve that is responsible for the limited application of big data tools in neuroimaging research, with only two published papers so far, one using Hadoop (Wang et al., 2013), the other using Spark (Freeman et al., 2014). The more tools are published to make the first steps with these technologies easier, of which the distributed NIfTI file reader provides a starting point, the more researchers will be able to use these tools, thus incentivizing further developments in this area. Compared to the software packages typically used by researchers in the field, Spark offers much simpler parallelization and scaling of analyses to arbitrarily large data sizes, but lacks most of the practical tools essential for convenient setup of analysis pipelines as they exist in more commonly used languages (i.e., python or R). Stronger links between these two worlds could allow for the development of analysis pipelines powerful enough to handle large datasets, yet as simple as any of the standard data applications.

### Conflict of interest statement

The authors declare that the research was conducted in the absence of any commercial or financial relationships that could be construed as a potential conflict of interest.
